# Alterations in cortical thickness and volumes of subcortical structures in pediatric patients with complete spinal cord injury

**DOI:** 10.1111/cns.14810

**Published:** 2024-06-18

**Authors:** Ling Wang, Beining Yang, Weimin Zheng, Tengfei Liang, Xin Chen, Qian Chen, Jubao Du, Jie Lu, Baowei Li, Nan Chen

**Affiliations:** ^1^ Department of Radiology and Nuclear Medicine, Xuanwu Hospital Capital Medical University Beijing China; ^2^ Beijing Key Laboratory of Magnetic Resonance Imaging and Brain Informatics Beijing China; ^3^ Department of Radiology, Beijing Chaoyang Hospital Capital Medical University Beijing China; ^4^ Department of Medical Imaging Affiliated Hospital of Hebei Engineering University Handan China; ^5^ Department of Radiology, Beijing Friendship Hospital Capital Medical University Beijing China; ^6^ Department of Rehabilitation Medicine, Xuanwu Hospital Capital Medical University Beijing China

**Keywords:** brain, complete spinal cord injury, cortical thickness, magnetic resonance imaging, pediatric

## Abstract

**Aims:**

To study the changes in cortical thickness and subcortical gray matter structures in children with complete spinal cord injury (CSCI), reveal the possible causes of dysfunction beyond sensory motor dysfunction after CSCI, and provide a possible neural basis for corresponding functional intervention training.

**Methods:**

Thirty‐seven pediatric CSCI patients and 34 age‐, gender‐matched healthy children as healthy controls (HCs) were recruited. The 3D high‐resolution T1‐weighted structural images of all subjects were obtained using a 3.0 Tesla MRI system. Statistical differences between pediatric CSCI patients and HCs in cortical thickness and volumes of subcortical gray matter structures were evaluated. Then, correlation analyses were performed to analyze the correlation between the imaging indicators and clinical characteristics.

**Results:**

Compared with HCs, pediatric CSCI patients showed decreased cortical thickness in the right precentral gyrus, superior temporal gyrus, and posterior segment of the lateral sulcus, while increased cortical thickness in the right lingual gyrus and inferior occipital gyrus. The volume of the right thalamus in pediatric CSCI patients was significantly smaller than that in HCs. No significant correlation was found between the imaging indicators and the injury duration, sensory scores, and motor scores of pediatric CSCI patients.

**Conclusions:**

These findings demonstrated that the brain structural reorganizations of pediatric CSCI occurred not only in sensory motor areas but also in cognitive and visual related brain regions, which may suggest that the visual processing, cognitive abnormalities, and related early intervention therapy also deserve greater attention beyond sensory motor rehabilitation training in pediatric CSCI patients.

## Graphical abstract

By exploring the changes in cortical thickness and subcortical gray matter structure after CSCI in children, we aim to reveal the possible causes of dysfunctions beyond sensory and motor deficits after CSCI, and provide a possible neural basis for corresponding functional intervention training.

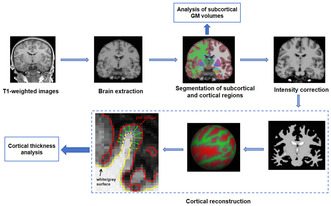

## 1 INTRODUCTION

Childhood is a critical period of development and maturation for individuals.^1^ Children at this stage are developing complicated reasoning skills, well visuospatial attention and demonstrating remarkable improvements in self‐regulating abilities, performing functions, and peer interactions.^1^, ^2^ The brain receives input from external stimulation and generates appropriate behavioral responses, and the cerebral cortex is in a dynamic process of development and maturation in this period.^3^, ^4^ After the initial thickening of the cortex in infancy and early childhood,^3^ cortical thinning occurs throughout the middle‐late childhood, adolescence, and even into the early adulthood.^4^, ^5^, ^6^ In addition, the deep gray matter (GM) nuclei form neural circuits with various brain regions of the cerebral cortex via nerve fibers, participating in various functions of the human body.^7^ Healthy brain development is very important for children's normal functioning, and aberrations in cortical and deep GM nuclei development may adversely affect cognition, emotion, behavior, or other functions.^8^

Traumatic complete spinal cord injury (CSCI) is characterized by sensory and motor disabilities and occurs in both adults and children, causing serious physical and psychological consequences.^9^ In children, CSCI may stunt brain development since they are in their golden years of development and maturation.^10^ Previous studies have shown that pediatric CSCI always presents many other physiological and psychological abnormalities, such as cognitive, emotional, behavioral, or other disorders, in addition to sensory motor dysfunction.^11^, ^12^ Similar to adult CSCI patients, the brain reorganization caused by the interruption of ascending and descending conduction pathways has been demonstrated to be a critical factor affecting the sensory motor rehabilitation of pediatric CSCI patients.^13^, ^14^ Nevertheless, it remains currently unclear whether there have been changes in the brain regions related to cognitive, emotional, or other functions after CSCI. Clarifying the changes in cerebral cortex and deep GM nuclei of children after CSCI may provide a possible neural basis for the cognitive‐emotional or other disorders beyond the sensory motor disabilities, and guide relevant early intervention treatment.

In the present study, we aimed to explore the effects of CSCI on children's cortical structure using cortical thickness, and also to investigate the effects of CSCI on children's subcortical structural volumes using 14 subcortical GM structures defined by the Desikan‐Killiany atlas.^15^ Furthermore, we examined the relationship between the altered brain structure and the motor scores, sensory scores, as well as the injury duration of the pediatric CSCI patients.

## 2 METHODS

### 2.1 Participants

This study was approved by the ethics committee of Xuanwu Hospital. Before undergoing study procedures, all parents or guardians of the subjects signed informed consent forms. Each pediatric CSCI patient was initially examined by two experienced physicians. The loss of consciousness assessment, post‐traumatic amnesia scale, and Glasgow coma scale were administered to exclude the possible loss of consciousness and post‐traumatic amnesia. Following that, the American Spinal Injury Association (ASIA) Impairment Scales (https://www.physio‐pedia.com/American_Spinal_Cord_Injury_Association_(ASIA)_Impairment_Scale) were assessed to evaluate the neurological status of the patients, including their sensory and motor functions. Patients included in this study should meet the following criteria: (1) have a history of trauma resulting in a diagnosis of CSCI; (2) aged 6–12 years; (3) with more duration than 2 months; (4) had no history of mental disorder or cognitive illness; (5) did not have any contraindication for MRI scanning; (6) right‐handedness. Healthy controls (HCs) enrolled in the present study were required to meet these criteria: (1) age and gender roughly matched those of the pediatric CSCI patients; (2) had no history of mental disorder or cognitive illness; (3) had no contraindication to MRI scanning; (4) right‐handedness.

### 2.2 MRI data collection

MRI scans were performed within 7 days after admission. All MRI data of the participants enrolled in this study were collected using a 12‐channel phase‐array head coil on a 3.0 T Siemens MRI scanner (Erlangen, Germany). Before the actual MRI examination, a mock MRI suite was used to ease children's concerns. Then, all participants were instructed to lie still and close their eyes. A pair of earplugs was used during the scan in order to reduce scanner noise, protecting the children's vulnerable auditory hair cells. The first step was to rule out any other cerebral abnormalities using the brain axial FLAIR (fluid‐attenuated inverse recovery) sequence, with the following parameters: 20 axial slices with a slice thickness = 5 mm, repetition time = 8000 ms, echo time = 94 ms, field of view = 240 × 240 mm^2^. Then, for each participant, high‐resolution T1‐weighted structural images were collected using a sagittal 3D magnetization‐prepared rapid gradient‐echo sequence (repetition time = 1800 ms; echo time = 2.13 ms; inversion time = 1100 ms; flip angle = 9 degrees; field of view = 256 × 256 mm; slice thickness = 1 mm; number of slices = 192; matrix = 256 × 256; voxel‐size = 1 × 1 × 1 mm^3^).

### 2.3 Data preprocessing

All images were reviewed by two radiologists with over 5‐year experience to rule out artifacts. Then, the T1‐weighted images were processed using the automated “recon‐all” processing pipeline of FreeSurfer (version 6.0, http://surfer.nmr.mgh.harvard.edu), which can generate a 3D model of the cortical surface and automatically segment the intensity values of the subcortical structures. The process mainly included motion correction, brain extraction, Talairach transform computation, segmentation of subcortical structures and cortical regions, intensity correction, and cortical reconstruction. The brain extraction step was performed by using Statistical Parametric Mapping (SPM) (Version 12; http://www.fil.ion.ucl.ac.uk/spm/software/spm12) according to a brain template for children aged 6–12 years (https://www.nitrc.org/projects/chn‐pd). Then, the GM, white matter (WM), and cerebrospinal fluid (CSF) were segmented, and the brain was divided into two hemispheres, each of which consisted of 40,962 vertices. Tessellation of the GM/WM boundary, as well as the inflation and registration of the cortical surface, were carried out. The cortex thickness was determined by computing the shortest distance between the gray‐white matter boundary and pial surface at each vertex. The surface maps were smoothed using a 10‐mm full‐width‐half‐maximum Gaussian kernel, and then were averaged across individuals by using a non‐rigid high‐dimensional spherical averaging method in order to align the cortical folding patterns.

The volumes of 14 subcortical gray matter structures defined by the Desikan‐Killiany atlas (including the bilateral hippocampus, amygdala, thalamus, nucleus accumbens, caudate nucleus, putamen, and pallidum) were acquired from an automated procedure for volumetric measurements implemented with FreeSurfer.

### 2.4 Statistical analysis

#### 2.4.1 Cortical thickness analysis

Differences in cortical thickness between pediatric CSCI patients and HCs were evaluated within the FreeSurfer's QDEC tool (https://surfer.nmr.mgh.harvard.edu/fswiki/Qdec). The group differences were tested in vertex‐wise maps by using a general linear model with age, gender, and average cortical thickness as covariates. Separate analyses were carried out on the right and left hemispheres. The resulting maps were corrected for multiple comparisons by using a Monte Carlo null‐*z* simulation (with 10,000 iterations). Clusters with a corrected cluster‐wise *p*‐value of less than 0.05 were considered significant.

#### 2.4.2 Analysis of subcortical GM volumes

Subcortical GM volumes were analyzed using SPSS software, version 22.0 (IBM, Armonk, NY, USA). All values were firstly analyzed for normality and homogeneity of variance, then two‐sample t‐tests were employed to compare differences for data with normal distribution and homogeneous variances, if not, Mann‐Whitney U tests would be used. The significance level was set at *p* < 0.05/14 = 0.0036 (14 comparisons for 14 subcortical GM structures defined by the Desikan‐Killiany atlas) with the Bonferroni correction method.

#### 2.4.3 Correlation analysis

The average cortical thickness and subcortical GM volume values of the brain regions with between‐group differences were extracted in each pediatric CSCI patient to analyze the correlation between these region's values and clinical features (such as the injury duration, sensory scores, and motor scores). Continuous variables were firstly analyzed for normality and homogeneity of variance, then partial correlation analyses were employed with age and gender as nuisance covariates to compare differences for data with normal distribution and homogeneous variances, if not, spearman correlation analyses would be used (*p* < 0.05, SPSS22.0).

## 3 RESULTS

### 3.1 Demographic and clinical characteristics

After three HCs with artifacts in their images were excluded, 37 pediatric thoracic CSCI patients (consisting of 5 male and 32 female patients; the mean age of patients was 8.51 ± 1.521 years, with an age range of 6–12 years) and 34 HCs (consisting of 5 males and 29 females; the mean age of HCs was 8.85 ± 2.091 years, with an age range of 6–12 years) were ultimately included in the present study. Disease duration of the patients ranged from 2 to 48 months, with a mean duration of 19.46 ± 13.353 months. No statistically significant difference was found between pediatric CSCI patients and HCs in age (Mann‐Whitney test, *p* = 0.544) or gender (Chi‐square test, *p* = 0.885). Detailed information on pediatric CSCI patients and HCs can be found in Table 1 and Table S1.

**TABLE 1:** Demographic and clinical data of pediatric CSCI patients and HCs.

Parameter	CSCI	HCs	*χ* ^2^, *t* or *z* value^a^	*p* value
Age	8.51 ± 1.521	8.85 ± 2.091	−0.607	0.544
Gender	M(5)/F(32)	M(5)/F(29)	0.021	0.885
Injury duration	19.46 ± 13.353	–	–	–
Mean cortical thickness_L (mm)	2.667 ± 0.070	2.677 ± 0.107	−1.301	0.193
Mean cortical thickness_R (mm)	2.665 ± 0.079	2.676 ± 0.104	0.476	0.635

Abbreviations: −, Not applicable; CSCI, complete spinal cord injury; F, female; HCs, healthy controls; L, left; M: male; R, right.

^a^ A positive *t* or *z* value indicates mean of pediatric CSCI patients is larger than that of HCs while a negative *t* or *z* value indicates mean of pediatric CSCI patients is less than that of HCs (all tests were two‐tailed).

### 3.2 Altered brain structure of pediatric CSCI patients

**FIGURE 1:**
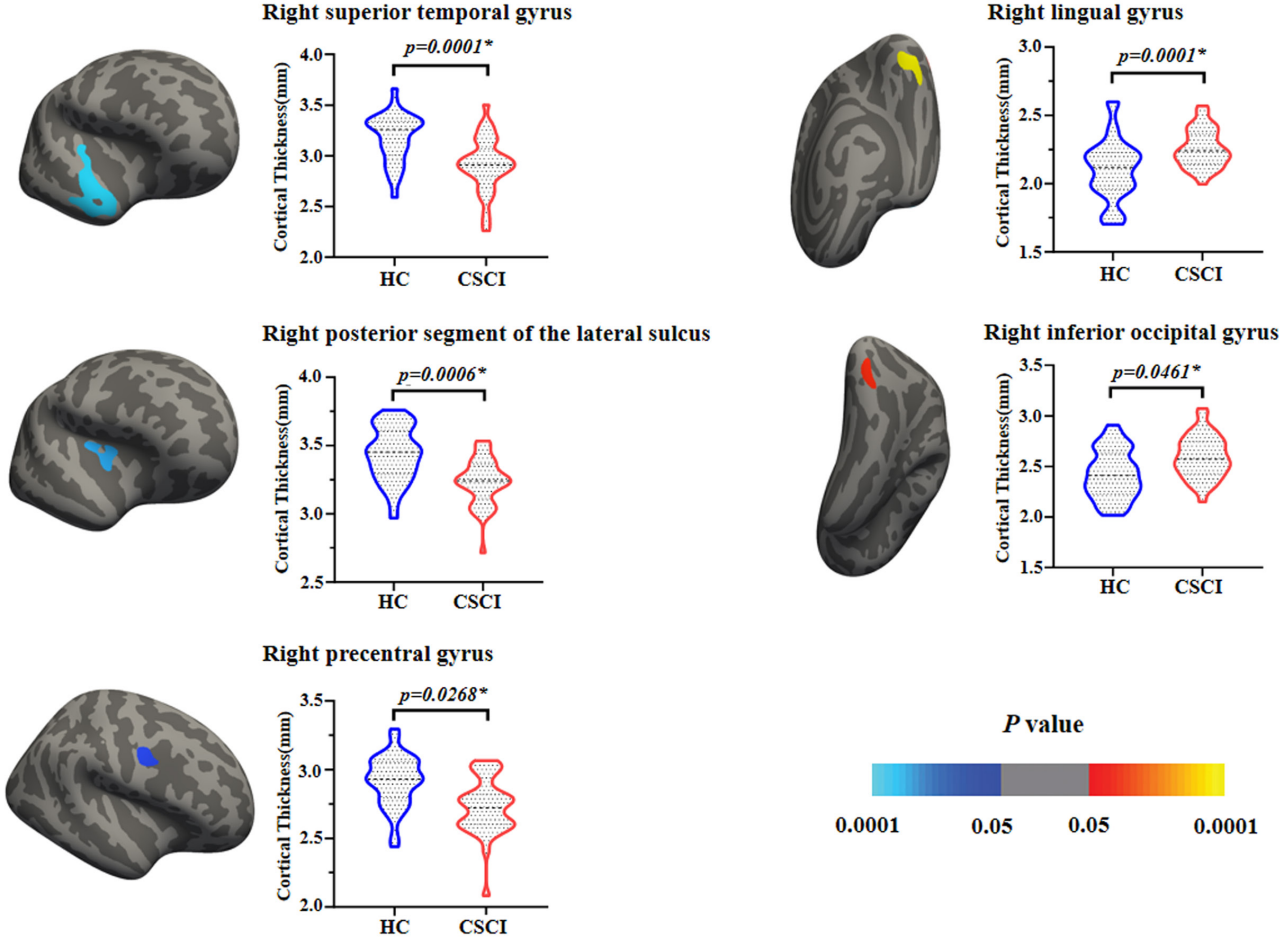
Differences in cortical thickness between the pediatric CSCI patients and HCs. Compared with HCs, pediatric CSCI patients showed significantly decreased cortical thickness in the right M1, STG, and posterior segment of the lateral sulcus, while increased cortical thickness in the right lingual gyrus and IOG (Corrected *p* < 0.05 for multiple comparisons, Monte Carlo null‐*z* simulation with 10,000 iterations). CSCI, complete spinal cord injury; HCs, healthy controls; IOG, inferior occipital gyrus; M1, precentral gyrus; STG, superior temporal gyrus.

There were no differences in mean cortical thickness between the patient group and the HCs in the left brain hemisphere (2.667 ± 0.070 mm vs. 2.677 ± 0.107 mm, *p*‐value = 0.193) or the right brain hemisphere (2.665 ± 0.079 mm vs. 2.676 ± 0.104 mm, *p*‐value = 0.635) (Table 1). In the cortical thickness analysis, pediatric CSCI patients showed decreased cortical thickness in the right precentral gyrus (M1), superior temporal gyrus (STG), and posterior segment of the lateral sulcus, while increased cortical thickness in the right lingual gyrus and inferior occipital gyrus (IOG) when compared with HCs (Table 2 and Figure 1).

**TABLE 2:** Brain regions showing cortical thickness alterations in pediatric CSCI patients.

Brain regions	MNI coordinate			Cluster size (mm^2^)	Peak *T* value
	*x*	*y*	*z*		
Regions with decreased cortical thickness in pediatric CSCI patients					
Right M1	54.7	2.1	33.5	235.19	−3.917
Right STG	55.8	3.3	−9.9	1213.29	−4.718
Right lateral sulcus	33.0	−20.1	9.1	419.93	−3.331
Regions with increased cortical thickness in pediatric CSCI patients					
Right lingual gyrus	11.9	−80.3	−5.3	553.31	3.896
Right IOG	39.2	−83.8	−7.1	211.94	2.932

Abbreviations: CSCI, complete spinal cord injury; IOG, inferior occipital gyrus; M1, precentral gyrus; STG, superior temporal gyrus.

**FIGURE 2:**
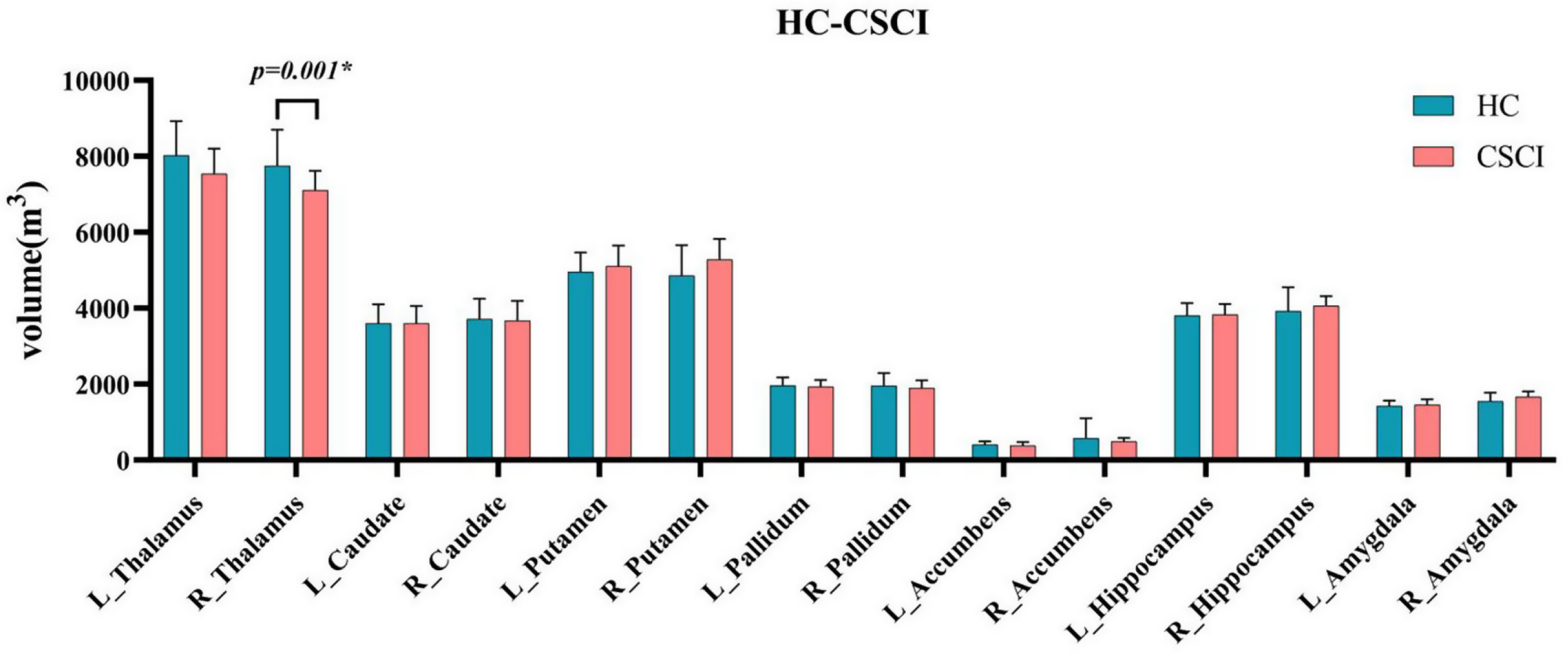
Volumes of the subcortical gray matter structures in the pediatric CSCI patients and HCs. Of the 14 subcortical gray matter structures, only the right thalamus showed a significant difference between the two groups. The volume of the right thalamus in pediatric CSCI patients was significantly smaller than that in HCs (Mann‐Whitney test, *p* = 0.001). CSCI, complete spinal cord injury; HCs, healthy controls; L, left; R, right.

Of the 14 subcortical gray matter structures selected for volumetric measurement, only the right thalamus showed a significant difference between the two groups (Mann‐Whitney test, *p* = 0.001). The volume of the right thalamus in pediatric CSCI patients was smaller than that in HCs (Figure 2).

### 3.3 Correlation analysis between altered brain structures and clinical characteristics in pediatric CSCI patients

No significant correlation was found between the cortical thickness values or the volume values in these altered brain regions and the clinical features of the patients, including the injury duration, sensory scores, and motor scores (*p* > 0.05).

## 4 DISCUSSION

Children with CSCI demonstrated alterations of cerebral structure in sensory motor related brain regions (decreased cortical thickness in the right M1 and decreased volume in the right thalamus), cognitive related brain regions (decreased cortical thickness in the right STG and posterior segment of the lateral sulcus), and visual related brain regions (increased cortical thickness in the right lingual gyrus and IOG) when compared with the HCs. Our findings suggest that CSCI can affect the brain structure of children, which might potentially explain the neural basis of the sensory motor, cognitive‐emotional, and visuospatial attention deficits in pediatric CSCI patients.

### 4.1 Structural brain alterations in sensory motor related brain regions

The present study demonstrated CSCI‐related cortical thinning in M1 and volume decreasing in thalamus. The M1 and thalamus play vital roles in sensory and motor functions through the cortico‐basal ganglia‐thalamo‐cortical loops.^16^ M1 is not only the target of output from the thalamus, basal ganglia, and cerebellum, but also the site where part of the corticospinal descending pathway originates.^17^, ^18^ It is located anterior to the central sulcus and is involved in motor learning, motor execution, and motor regulation.^17^, ^19^ Decreased cortical thickness in M1 may be contributed by smaller arborizations per neuron, decreased glial volume, or decreased regional vasculature.^20^ As a key node of the cortico‐basal ganglia‐thalamo‐cortical loops, the thalamus is an important structure that transmits and processes sensory and motor signals to the cerebral cortex.^16^ It plays a crucial role in relaying and integrating sensory motor information between the cortex and other subcortical structures.^16^, ^21^ Our findings might support the view that CSCI could cause significant anatomical atrophy in children's cortico‐basal ganglia‐thalamo‐cortical loops, resulting in abnormal sensory and motor functions.

### 4.2 Structural brain alterations outside sensory motor related brain regions

We also found CSCI‐related cortical thinning in the right STG and posterior segment of the lateral sulcus (the insula), which are involved in the cognitive process.^22^, ^23^, ^24^, ^25^, ^26^, ^27^ The STG has been shown to be the last to fully mature during the childhood.^22^, ^23^ It is mainly responsible for cognitive functions and social interactions, playing a key role in perceiving emotions from language function, auditory processing, facial stimuli, and social cognition.^24^, ^25^ The posterior segment of the lateral sulcus is composed of the superior and inferior segments of the circular sulcus of the insula.^26^ As a hub region of the salience network, the insula is primarily involved in perception, emotion, and higher order cognitive functions.^27^ Although we were not able to examine the associations between the right STG and insular cortical thinning and the cognitive functions of pediatric CSCI patients in the current study, previous studies have shown that changes in cortical structure may impact the brain functions,^28^, ^29^ and we, therefore, speculate that children may be at risk for poor cognitive development after CSCI.

In addition, we found increased cortical thickness in the right lingual gyrus and IOG, which are mainly involved in visual processes. The lingual gyrus is not only responsible for visual associations and episodic memory consolidation, but also a key component of the network related to verbal declarative memory.^30^ In addition, it is a part of the color sensitive visual region V4, and seems to be sensitive to both spatial and feature based attention.^31^ The IOG is the most posterior region of the brain and one of the core human neural systems for facial perception, it correlates with the initial stage of face processing.^32^ Previous scholars have demonstrated a critical role for vision in all aspects of movement, including motor planning, movement control, error evaluation, and feedback.^33^, ^34^, ^35^ Deficits in visuospatial neural functions may affect the motor performance of children.^36^ Moreover, children's vision may also be impacted by differences in motor skill acquisition since visuospatial attention has been demonstrated to develop with locomotor experience in early childhood.^37^, ^38^, ^39^ Individuals with sensory motor dysfunctions may require precise information from their visual environment to adapt their movements and complete functional tasks like reaching.^33^, ^38^ Development with limbs' sensory motor dysfunctions in pediatric SCI patients may impact visuospatial attention, leading to changes in cortical thickness of visual related brain regions. As suggested by earlier literature, widespread progressive decreases in cortical thickness of the typical developmental children usually occur over time due to the programmed apoptosis.^40^ Increased cortical thickness in lingual gyrus and IOG might indicate the disruption of brain development, or it may also suggest that extensive plastic processes in visual related regions might occur to compensate for the sensory motor dysfunctions in children after CSCI. The specific neural mechanism remains to be studied in the future.

### 4.3 Limitations

In this study, several limitations are present. Firstly, this study was cross‐sectional, and a future longitudinal study will be necessary to reveal how the brain reorganizes with the progression of the disease. Secondly, the majority of the patients enrolled in the present study were girls, and most of them had a clear traumatic history after backbend during dance practice. More pediatric CSCI patients will be recruited in future studies to further validate these findings. Thirdly, we did not acquire any data on mood, cognition, or vision in these pediatric patients, so their associations with our findings could not be explored. The behavioral correlates of these brain regions should be investigated in future studies among pediatric CSCI patients to provide a more comprehensive understanding of their relationship.

## 5 CONCLUSION

This study provides additional findings that can help further explain the brain structural reorganization of pediatric CSCI patients, contributing to our understanding of the effects of CSCI on the neurodevelopment of children. In addition to the reorganization of sensory motor related brain regions, we also found alterations in cognitive related brain regions and visual related areas. We contend that the visual processing and cognitive abnormalities in pediatric CSCI patients also deserve greater attention, as these deficits may negatively impact children's social cognitive functioning and quality of life.

## AUTHOR CONTRIBUTIONS

Ling Wang contributed to study design, data acquisition and analysis, the manuscript draft; Beining Yang contributed to study design, data acquisition; Weimin Zheng contributed to data acquisition, critical revision of the manuscript; Tengfei Liang contributed to data analysis and interpretation; Xin Chen and Qian Chen contributed to data acquisition and statistical analysis; Jubao Du and Jie Lu contributed to study design and data acquisition; Baowei Li contributed to critical revision of the manuscript for important content; Nan Chen contributed to study concept and design, data interpretation, manuscript revision, study supervision, funding acquisition. All authors read and approved the final manuscript.

## FUNDING INFORMATION

This work was supported by the National Natural Science Foundation of China [grant numbers 81871339, 81271556]; the Beijing Municipal Natural Science Foundation [grant number 7113155]; and the Science Foundation of Beijing Municipal Commission of Education [grant number KM201210025013].

## CONFLICT OF INTEREST STATEMENT

The authors declare no financial or other conflicts of interest.

## Supporting information


Table S1.

## Data Availability

The datasets generated for this study are available on request to the corresponding authors.
