# Supplementary material for: Alterations in cortical thickness and volumes of subcortical structures in pediatric patients with complete spinal cord injury
Source: CNS Neurosci Ther. 2024 Jun 18;30(6):e14810. doi: 10.1111/cns.14810 (PMC11183907; doi:10.1111/cns.14810)
Supplement: Supplementary file 1 — Table S1. [file CNS-30-e14810-s001.docx]

**Table S1 Clinical data of pediatric CSCI patients.**

| Subjects | Age  (years) | Gender | Etiology | Duration  (months) | Level of lesion | ASIA | Motor  Scores  (0-100) | Pinprick sensory  Scores (0-224) | Light touch sensory  Scores (0-224) |
| --- | --- | --- | --- | --- | --- | --- | --- | --- | --- |
| 1 | 8 | F | Backbend^a^ | 30 | T11 | A | 50 | 70 | 70 |
| 2 | 10 | F | Backbend | 28 | T10 | A | 50 | 64 | 64 |
| 3 | 8 | F | Backbend | 33 | T8 | A | 50 | 60 | 60 |
| 4 | 7 | F | Fall | 28 | T7 | A | 50 | 56 | 56 |
| 5 | 8 | F | Backbend | 2 | T7 | A | 50 | 52 | 52 |
| 6 | 12 | F | Backbend | 24 | T10 | A | 50 | 64 | 64 |
| 7 | 9 | F | Backbend | 19 | T9 | A | 50 | 64 | 64 |
| 8 | 11 | F | Backbend | 48 | T9 | A | 50 | 64 | 64 |
| 9 | 6 | M | Traffic accident | 3 | T10 | A | 50 | 64 | 64 |
| 10 | 11 | F | Backbend | 36 | T9 | A | 50 | 64 | 64 |
| 11 | 10 | F | Backbend | 14 | T9 | A | 50 | 72 | 72 |
| 12 | 9 | F | Backbend | 12 | T8 | A | 50 | 64 | 64 |
| 13 | 10 | F | Fall | 22 | T7 | A | 50 | 60 | 60 |
| 14 | 10 | F | Backbend | 5 | T8 | A | 50 | 64 | 64 |
| 15 | 7 | F | Backbend | 9 | T10 | A | 50 | 64 | 64 |
| 16 | 8 | M | Sprain | 4 | T7 | A | 50 | 44 | 44 |
| 17 | 9 | F | Backbend | 14 | T9 | A | 50 | 64 | 64 |
| 18 | 8 | F | Backbend | 16 | T9 | A | 50 | 64 | 64 |
| 19 | 6 | F | Backbend | 14 | T9 | A | 50 | 64 | 64 |
| 20 | 9 | M | Fall | 35 | T9 | A | 50 | 64 | 64 |
| 21 | 9 | F | Backbend | 42 | T2 | A | 50 | 40 | 40 |
| 22 | 10 | F | Backbend | 47 | T9 | A | 50 | 60 | 60 |
| 23 | 9 | F | Backbend | 21 | T10 | A | 50 | 72 | 72 |
| 24 | 7 | M | Traffic accident | 46 | T3 | A | 50 | 76 | 76 |
| 25 | 11 | F | Fall | 36 | T8 | A | 50 | 56 | 56 |
| 26 | 9 | F | Backbend | 8 | T9 | A | 50 | 62 | 62 |
| 27 | 7 | F | Backbend | 5 | T10 | A | 50 | 70 | 70 |
| 28 | 7 | F | Traffic accident | 7 | T5-8 | A | 50 | 60 | 60 |
| 29 | 7 | F | Backbend | 12 | T9 | A | 50 | 64 | 64 |
| 30 | 10 | F | Backbend | 12 | T11 | A | 50 | 88 | 88 |
| 31 | 8 | F | Backbend | 11 | T5 | A | 50 | 44 | 44 |
| 32 | 9 | F | Backbend | 18 | T10-12 | A | 50 | 68 | 68 |
| 33 | 7 | M | Fall | 21 | T9 | A | 50 | 60 | 60 |
| 34 | 7 | F | Backbend | 11 | T11 | A | 50 | 70 | 70 |
| 35 | 7 | F | Backbend | 8 | T9-10 | A | 50 | 72 | 72 |
| 36 | 8 | F | Backbend | 9 | T10 | A | 50 | 70 | 68 |
| 37 | 7 | F | Backbend | 10 | T8-9 | A | 50 | 70 | 66 |

Note: The level of lesion refers to the neurological level. ASIA: American Spinal Injury Association, ASIA impairment scale: A: complete—no sensory or motor function is preserved in sacral segments S4–S5, sensory score: sum of segmental light touch and pinprick classifications. Backbend^a^: pediatric patients had a clear traumatic history after backbend during dance practice. CSCI: complete spinal cord injury, F: female, M: male.
